# Supplementary material for: TransferGWAS of T1-weighted brain MRI data from UK Biobank
Source: PLoS Genet. 2024 Dec 13;20(12):e1011332. doi: 10.1371/journal.pgen.1011332 (PMC11687923; doi:10.1371/journal.pgen.1011332)
Supplement: S1 Text — Fig A: Distribution of the p-values from the GWAS from ADNI and ImageNet model training. Fig B: Venn diagrams of the number of discovered loci on each chromosome. Fig C: Results of the phenome-wide association study between the polygenic scores fitted on the features of the deep learning models (rows) and phenotypes from the UK Biobank dataset. Fig D: Results of the PheWAS between the polygenic scores fitted on the features of the DL models (rows) and phenotypes from the UK Biobank dataset. Fig E: Difference images for the brain MRI scans sorted according to their corresponding values of the principal components (PCs) of the ADNI trained model. Fig F: Difference images for the brain MRI scans sorted according to their corresponding values of the PCs of the ImageNet trained model. Fig G: Statistical parametric mappings (SPMs) for the first 10 principal components of the model trained on the ADNI dataset. Fig H: Statistical parametric mappings (SPMs) for the first 10 principal components of the model trained on the ImageNet dataset. Fig I: Ratio of variance explained in each brain region of interest (ROI) by each principal component of the neural network models features. Table A: Comparison of predictive performance of Multi-PGS models on the full UKB sample. Table B: Pearson correlation coefficients of each of the 10 PCs of the ADNI-trained variational autoencoder (VAE) model and age and sex covariates on the UKB data. (PDF) [file pgen.1011332.s001.pdf]

List of Figures

A **Distribution of the p-values from the GWAS from ADNI and ImageNet model training.** Shown are quantile-quantile plots of observed p-values for each principal component of the ADNI-trained model (first two rows) and the ImageNet-trained model (last two rows). 2

B **Venn diagrams of the number of discovered loci on each chromosome** discovered by either the ADNI model (red), ImageNet model (green), or both (beige). 3

C **Results of the phenome-wide association study (PheWAS) (white British sample, $N = 424,609$) between the polygenic scores (PGS) fitted on the features of the deep learning (DL) models (rows) and phenotypes from the UK Biobank (UKB) dataset (columns).** Cell colors represent the magnitudes and the signs of the estimated association coefficients between each PGS and phenotype combination. 4

D **Results of the PheWAS (full UKB sample, $N = 451,450$) between the polygenic scores (PGS) fitted on the features of the DL models (rows) and phenotypes from the UK Biobank (UKB) dataset (columns).** Cell colors represent the magnitudes and the signs of the estimated association coefficients between each PGS and phenotype combination. 5

E **Difference images for the brain MRI scans sorted according to their corresponding values of the principal components (PCs) of the ADNI trained model.** Scans are grouped into 9 percentiles according to the PC values and averaged into a single scan. The scan from the middle percentile is then subtracted from the lower and upper percentiles images to create heatmaps indicating brain changes leading to negative (left columns) or positive (right columns) values of the PCs. 8

F **Difference images for the brain MRI scans sorted according to their corresponding values of the PCs of the ImageNet trained model.** Scans are grouped into 9 percentiles according to the PC values and averaged into a single scan. The scan from the middle percentile is then subtracted from the lower and upper percentiles images to create heatmaps indicating brain changes leading to negative (left columns) or positive (right columns) values of the PCs. 10

G **Statistical parametric mappings (SPMs) for the first 10 principal components of the model trained on the ADNI dataset.** Plotted are values of the t-statistics of the statistically significant correlation coefficients between between each PC and each single voxel in the MRI scans. We plot values below the Bonferroni-corrected significance threshold accounting for the total number of voxels tested. . 12

H **Statistical parametric mappings (SPMs) for the first 10 principal components of the model trained on the ImageNet dataset.** Plotted are values of the t-statistics of the statistically significant correlation coefficients between between each PC and each single voxel in the MRI scans. We plot values below the Bonferroni-corrected significance threshold accounting for the total number of voxels tested. . 13

I **Ratio of variance explained in each brain region of interest (ROI) by each principal component of the neural network models features**. Values are computed as an average ratio over all voxels belonging to a ROI. Shown are only the pairs of PC – ROI with a ratio of explained variance higher than 0.01. 14

List of Tables

A **Comparison of predictive performance of Multi-PGS models on the full UKB sample** using only trait-specific polygenic scores (PGS) (2nd column) and including our TransferGWAS PGS (3rd column) for a set of selected phenotypes from UK Biobank (UKB), measured with the R^2 coefficient of determination. Significant differences are marked with (*). Heel bone mineral density (1) and (2) correspond to results of using PGS for heel bone mineral density, or (general) bone mineral density respectively. Statistical significance was estimated using pairwise permutation tests with $1,000$ permutations. 5

B **Pearson correlation coefficients of each of the 10 PCs of the ADNI-trained variational autoencoder (VAE) model and age and sex covariates on the UKB data.** 14

1 Additional GWAS Plots

1.1 Quantile-quantile Plots

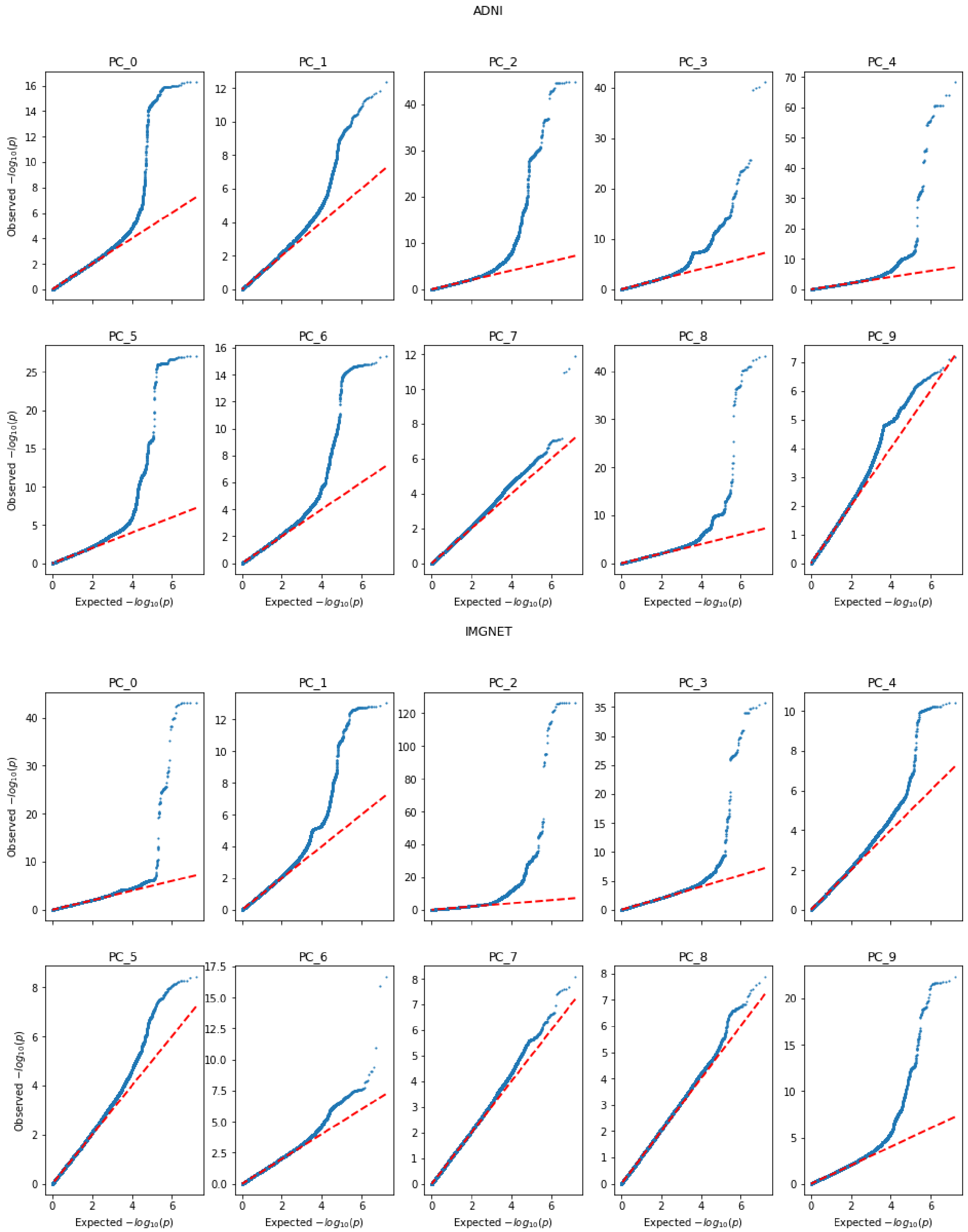

Fig A: **Distribution of the p-values from the GWAS from ADNI and ImageNet model training.** Shown are quantile-quantile plots of observed p-values for each principal component of the ADNI-trained model (first two rows) and the ImageNet-trained model (last two rows).

1.2 Number of Loci Shared Between the Two Models per-chromosome

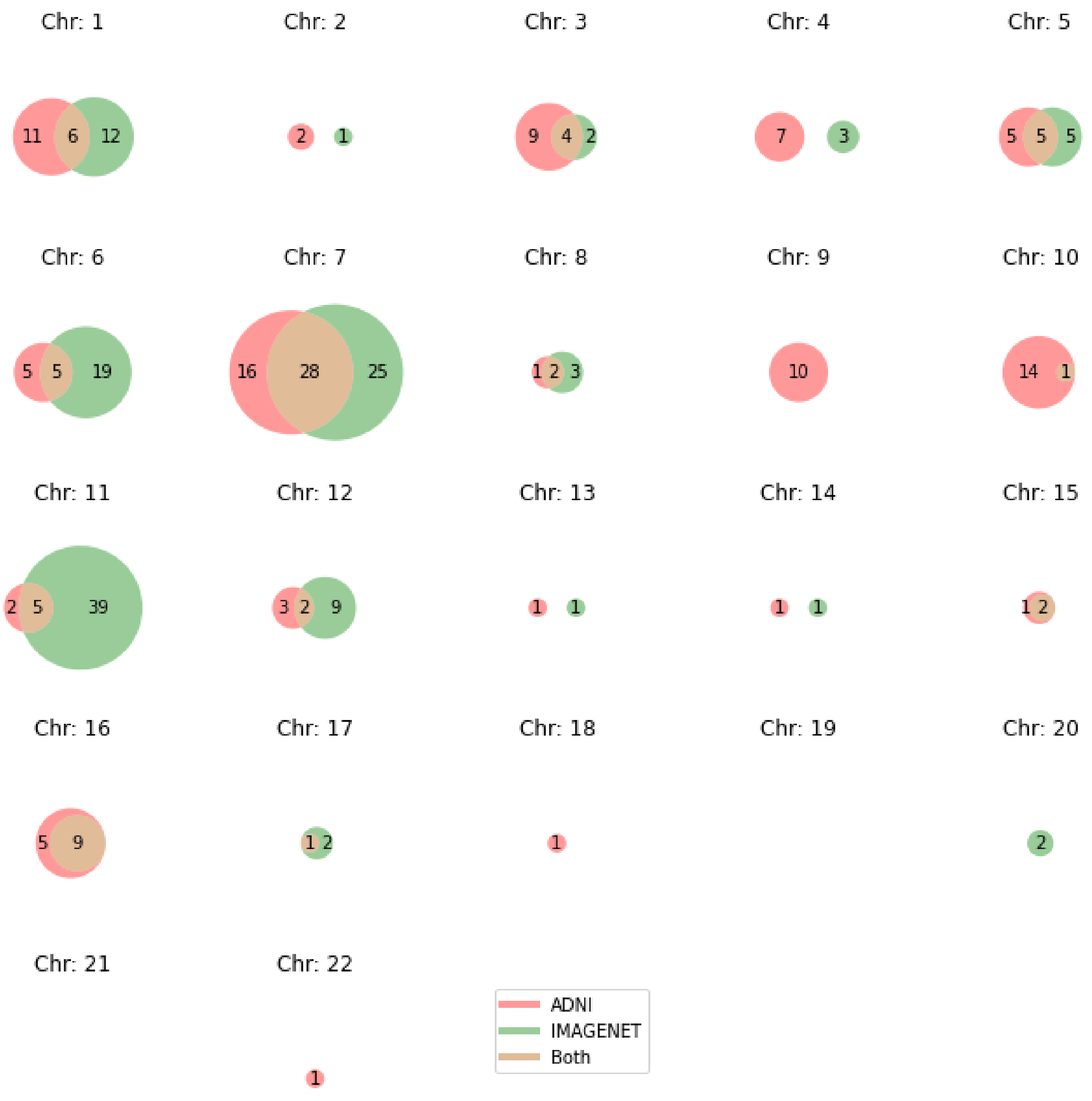

Fig B: **Venn diagrams of the number of discovered loci on each chromosome** discovered by either the ADNI model (red), ImageNet model (green), or both (beige).

2 Polygenic Scores

2.1 PheWAS

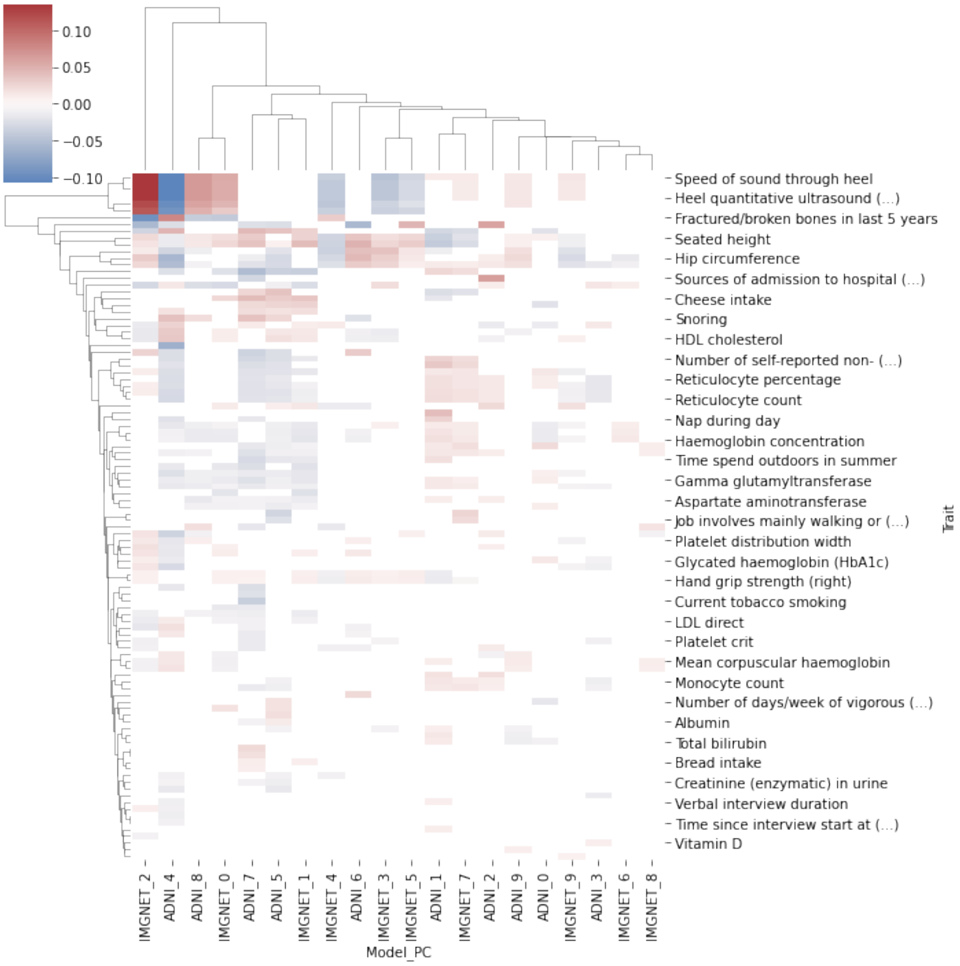

Fig C: **Results of the PheWAS (white British sample, $N = 424,609$) between the polygenic scores (PGS) fitted on the features of the DL models (rows) and phenotypes from the UK Biobank (UKB) dataset (columns).** Cell colors represent the magnitudes and the signs of the estimated association coefficients between each PGS and phenotype combination.

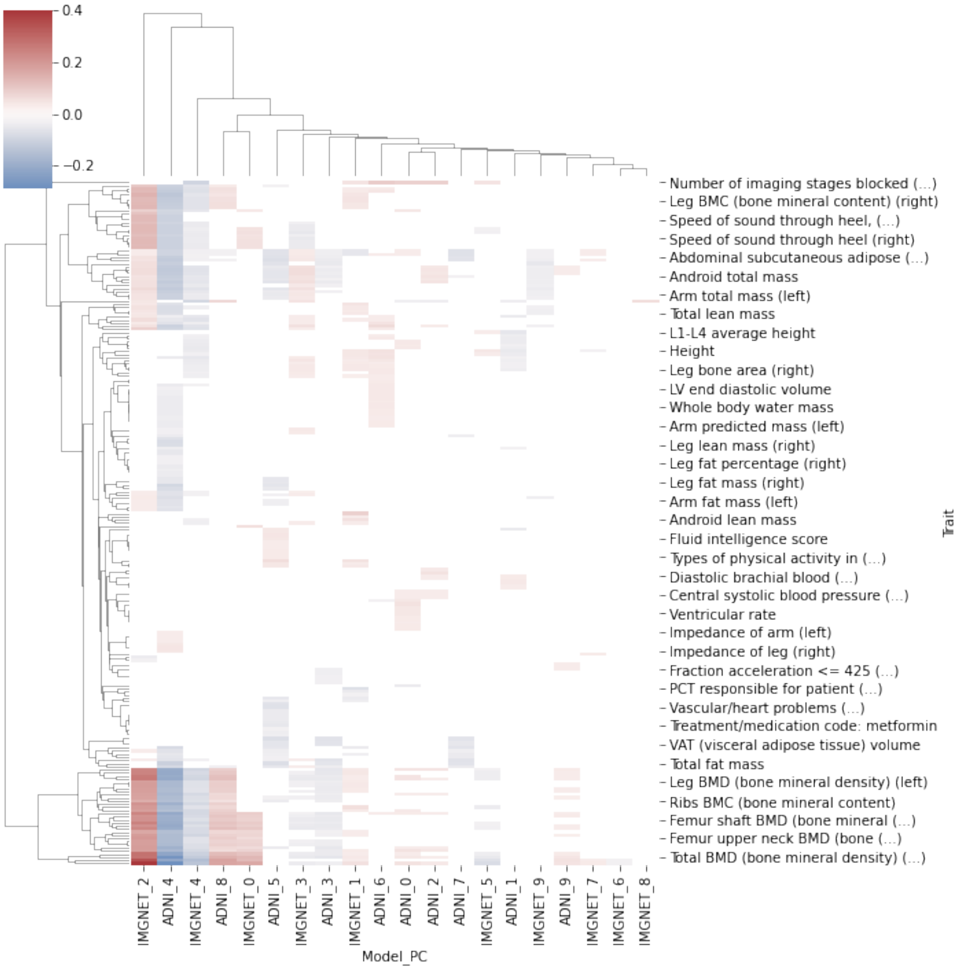

Fig D: **Results of the PheWAS (full UKB sample, $N = 451,450$) between the polygenic scores (PGS) fitted on the features of the DL models (rows) and phenotypes from the UK Biobank (UKB) dataset (columns).** Cell colors represent the magnitudes and the signs of the estimated association coefficients between each PGS and phenotype combination.

2.2 Predictive Evaluation

Trait	PGS Catalog	Our PGS + PGS Catalog	Δ
Height	0.759	0.759	0.000
BMI	0.284	0.284	0.000
Heel bone mineral density (1)	0.273	0.275	0.002*
Heel bone mineral density (2)	0.064	0.078	0.014*
Red blood cell count	0.387	0.387	0.000
White blood cell count	0.104	0.106	0.002*
Systolic blood pressure	0.283	0.284	0.000
Diastolic blood pressure	0.175	0.176	0.001*
Ventricular rate	0.027	0.025	-0.001

Table A: **Comparison of predictive performance of Multi-PGS models on the full UKB sample** using only trait-specific polygenic scores (PGS) (2nd column) and including our TransferGWAS PGS (3rd column) for a set of selected phenotypes from UK Biobank (UKB), measured with the R^2 coefficient of determination. Significant differences are marked with (*). Heel bone mineral density (1) and (2) correspond to results of using PGS for heel bone mineral density, or (general) bone mineral density respectively. Statistical significance was estimated using pairwise permutation tests with 1,000 permutations.

3 Additional Interpretation Analyses of the DNN Features

3.1 Per-PC Difference Images

For each PC of the ADNI and ImageNet trained deep neural network (DNN) models we sorted the corresponding scans from UKB according to the values of the PC, grouped them in 9 percentiles of equal size, and computed the average scan per percentile. We assumed the average scan from the middle percentile to serve as a "baseline" image, and subtracted it from the average scans from the remaining 8 percentiles, corresponding to the 4 percentiles with negative and 4 percentiles with positive PC values. This allowed us to investigate what changes in the brain structures lead to an increase of the PC values in the negative and positive directions. We plotted the difference images for the middle slice for each of the 3 brain MRI axes in Figure E for the ADNI and Figure F for the ImageNet trained models respectively. PCs 2 of the ADNI model and 0 and 1 of the ImageNet model seem to reflect the overall intensity of the scans, while certain PCs focus on more specific brain structures, e.g., the lateral ventricles (PCs 1,3,4,6,7,8 of the ADNI and 2,3,4 of the ImageNet models). We note that the ImageNet PCs 3-10 seem to exhibit a non-linear relationship wrt. the brain MRI voxels, compared to the ADNI trained VAE model.

PC 0

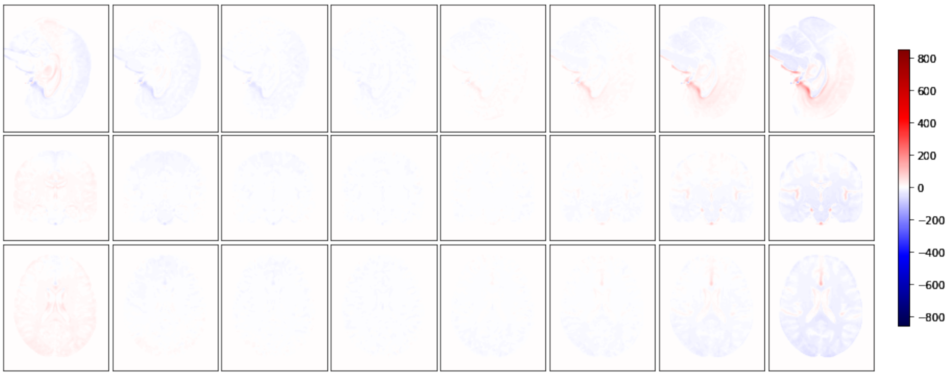

PC 1

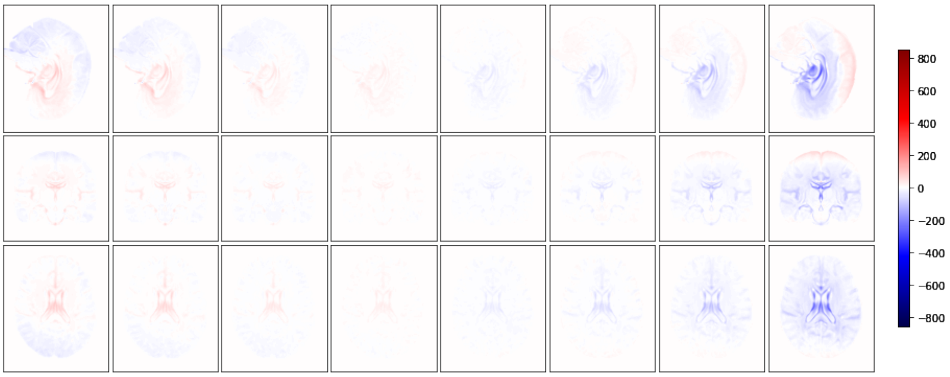

PC 2

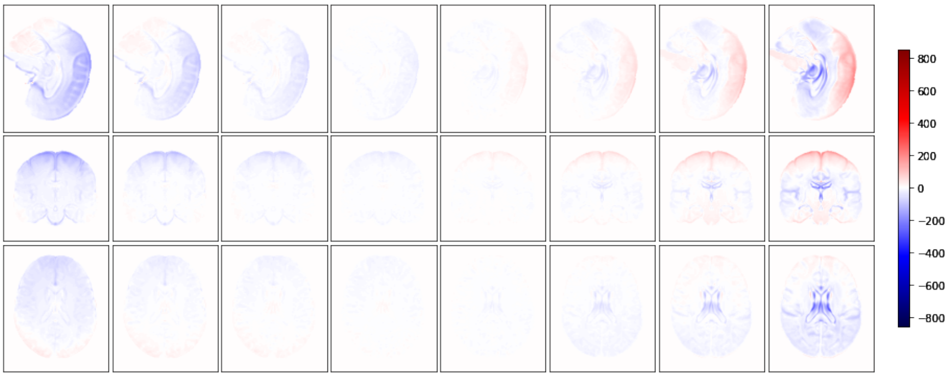

PC 3

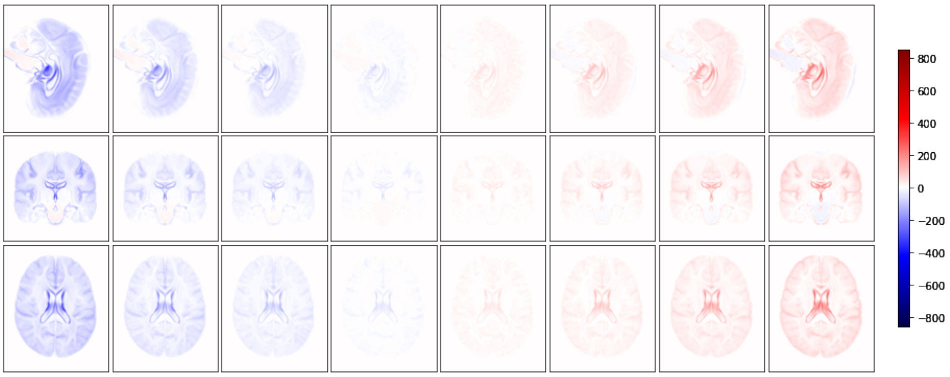

PC 4

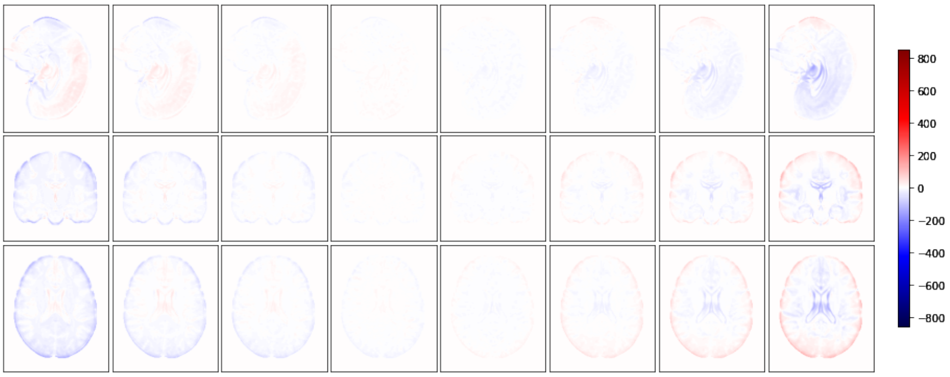

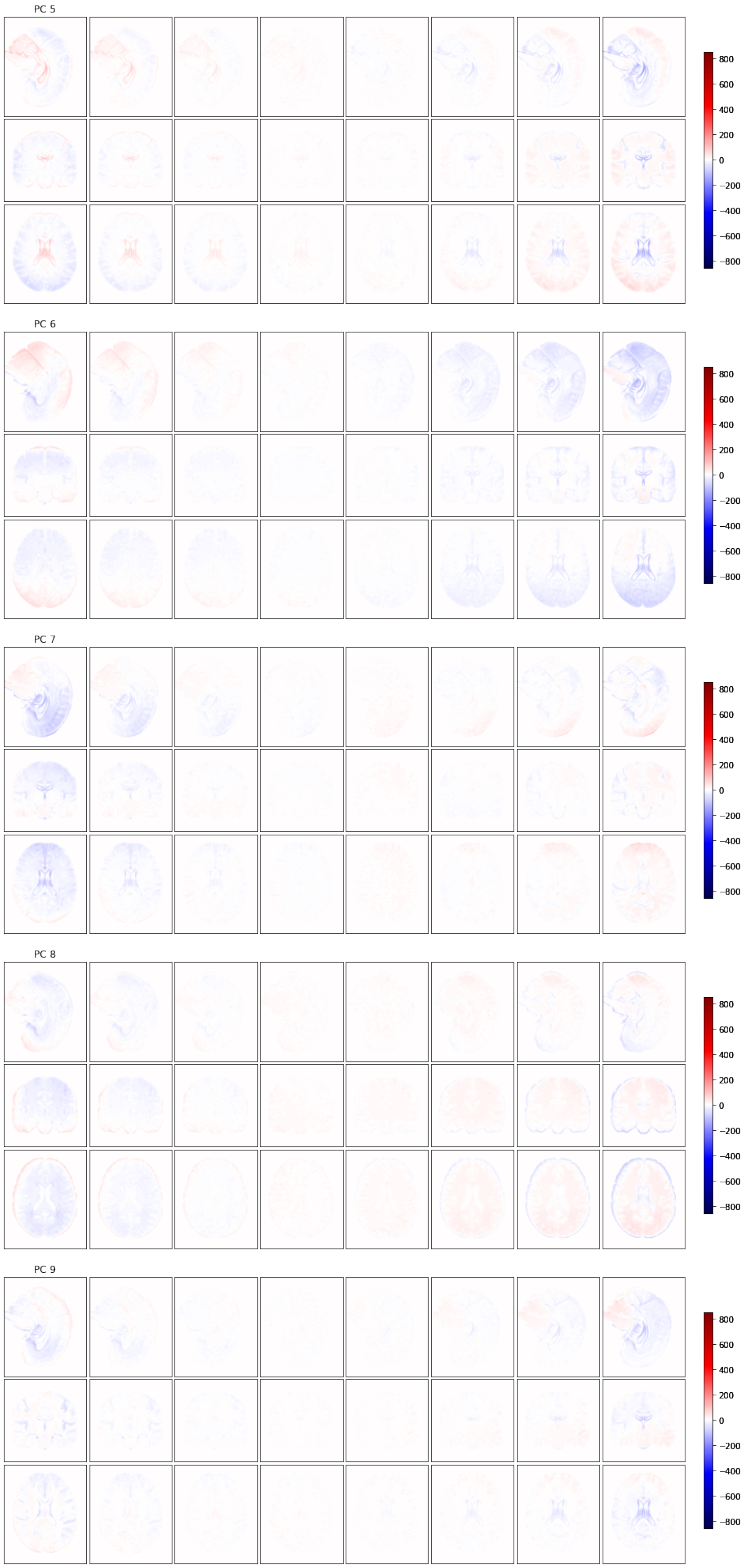

Fig E: **Difference images for the brain MRI scans sorted according to their corresponding values of the PCs of the ADNI trained model.** Scans are grouped into 9 percentiles according to the PC values and averaged into a single scan. The scan from the middle percentile is then subtracted from the lower and upper percentiles images to create heatmaps indicating brain changes leading to negative (left columns) or positive (right columns) values of the PCs.

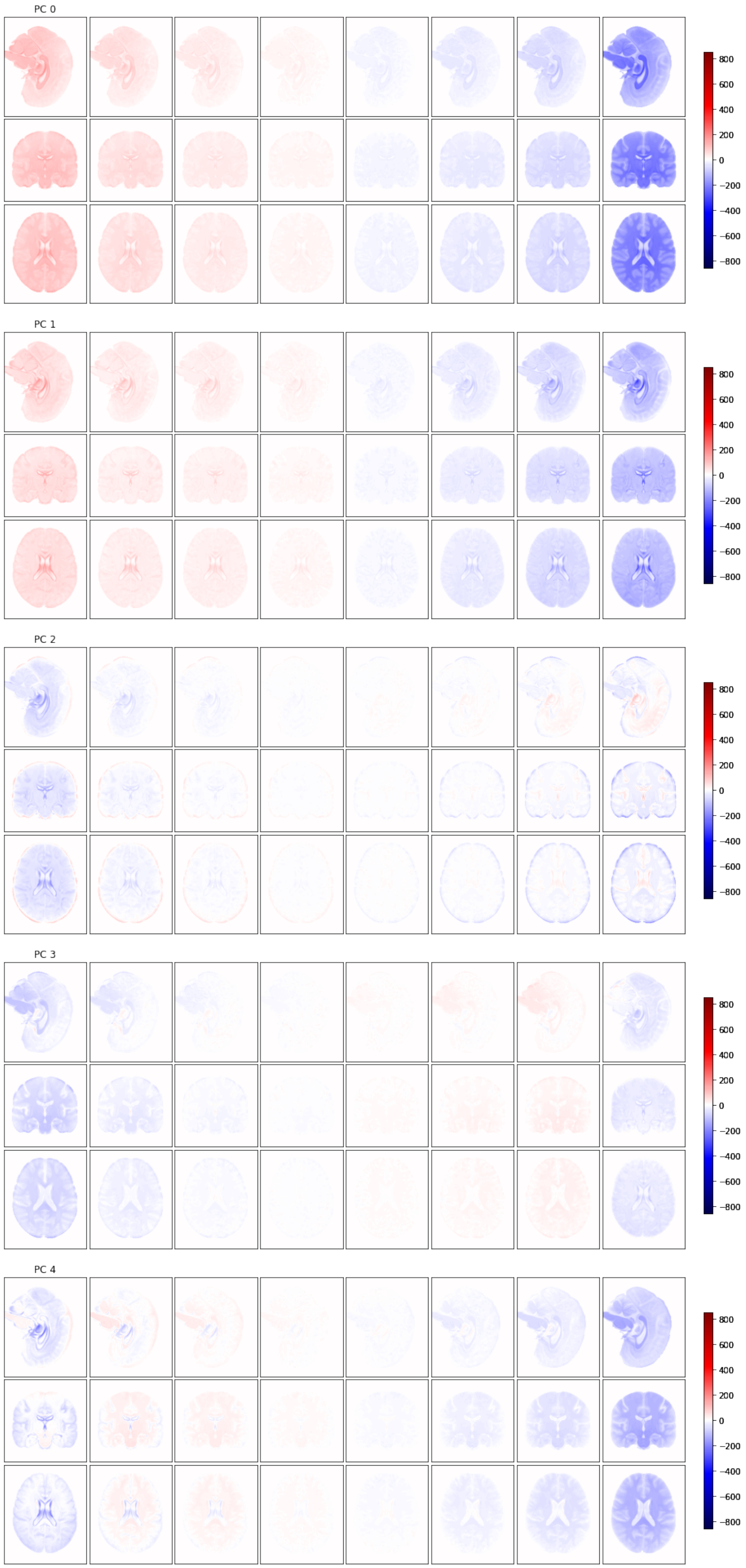
PC 0
PC 1
PC 2
PC 3
PC 4
800
600
400
200
0
−200
−400
−600
−800

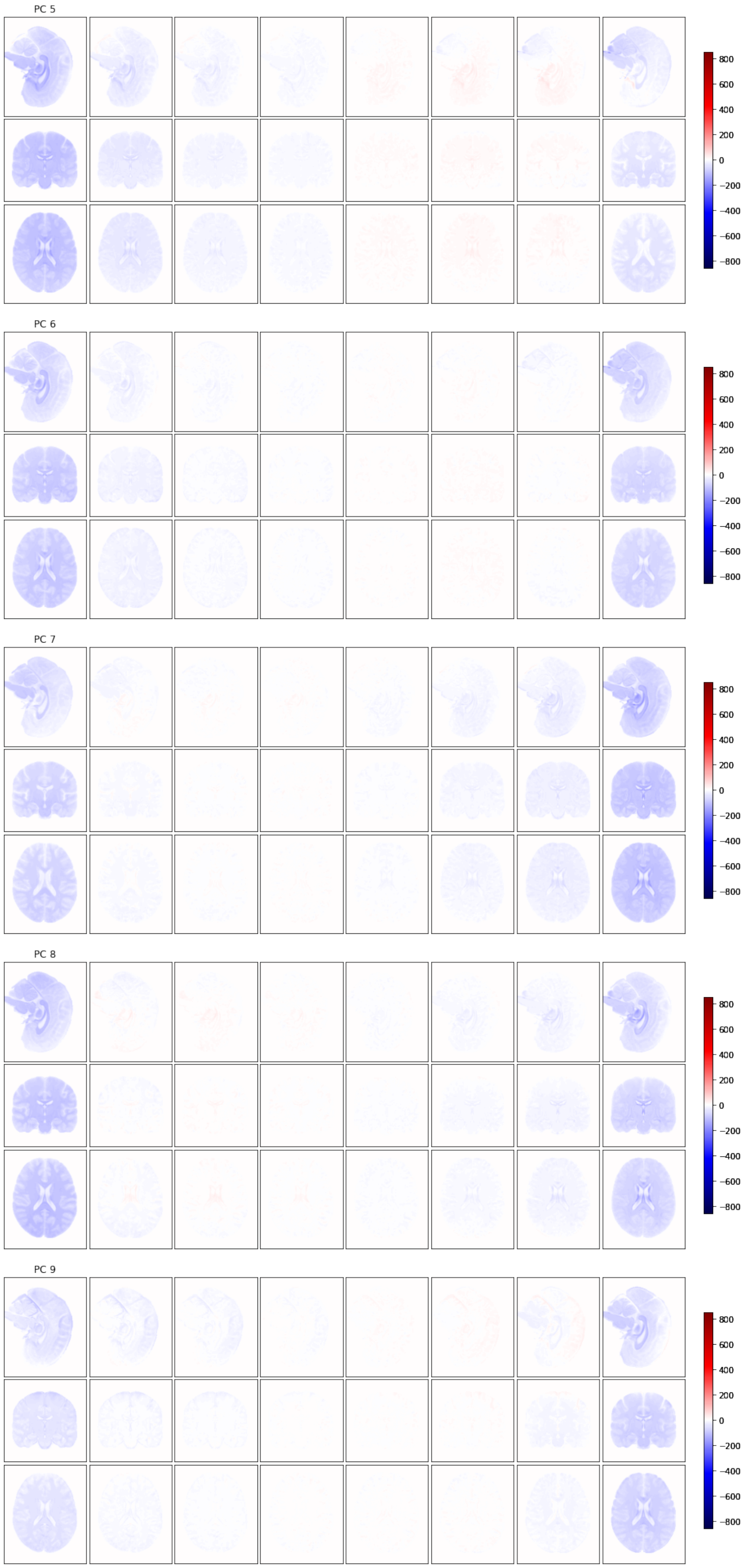

Fig F: **Difference images for the brain MRI scans sorted according to their corresponding values of the PCs of the ImageNet trained model.** Scans are grouped into 9 percentiles according to the PC values and averaged into a single scan. The scan from the middle percentile is then subtracted from the lower and upper percentiles images to create heatmaps indicating brain changes leading to negative (left columns) or positive (right columns) values of the PCs.

3.2 Statistical Parametric Mappings

We computed statistical parametric mappings (SPMs) for each of the 10 PCs for the ADNI (Figure E) and ImageNet (Figure H) trained models. We plotted values of the t-statistics of the statistically significant correlation coefficients between each PC and each single voxel in the MRI scans. The intensity of the voxels can thus be interpreted as the magnitude of correlation between a PC and a voxel.

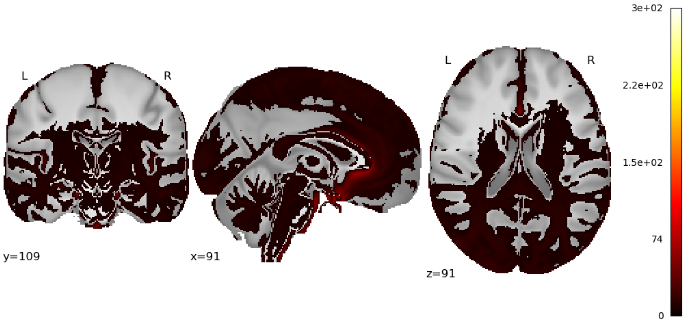

(a) PC 0

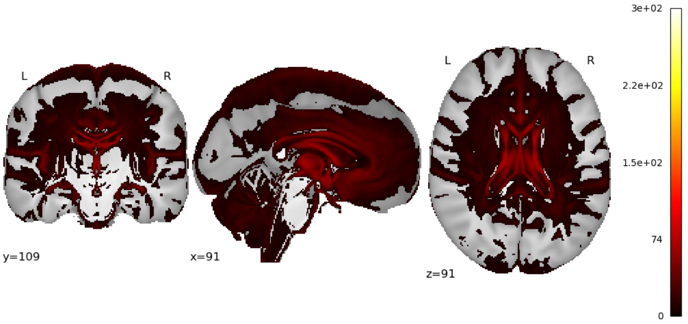

(b) PC 1

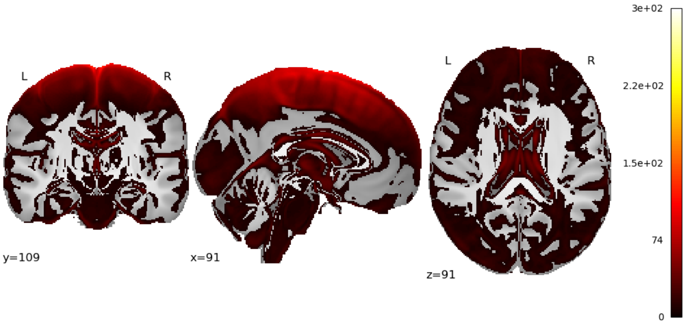

(c) PC 2

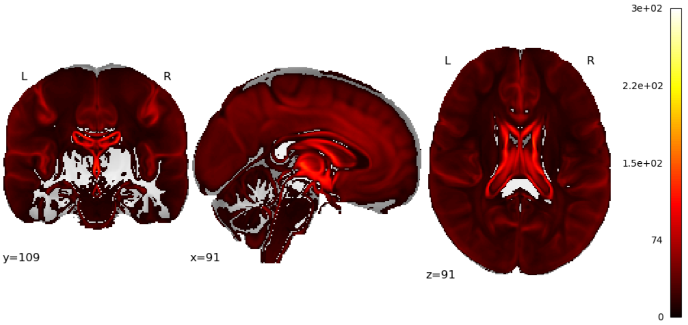

(d) PC 3

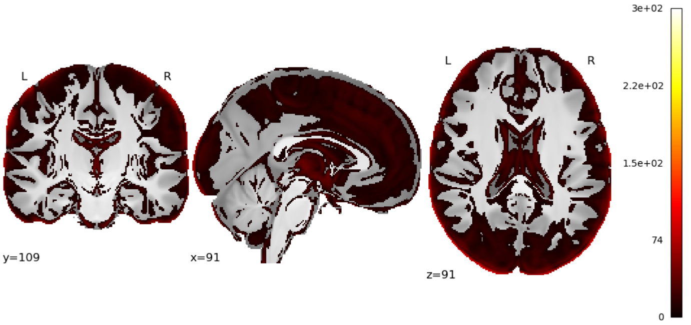

(e) PC 4

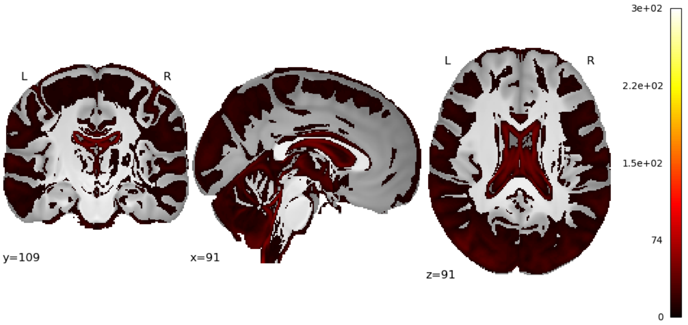

(f) PC 5

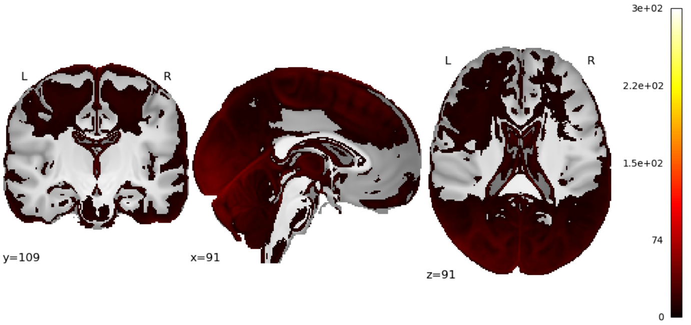

(g) PC 6

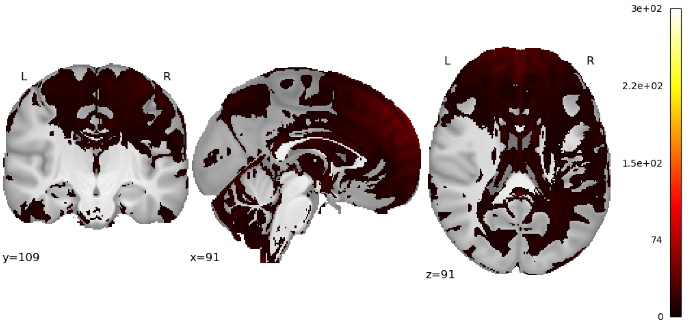

(h) PC 7

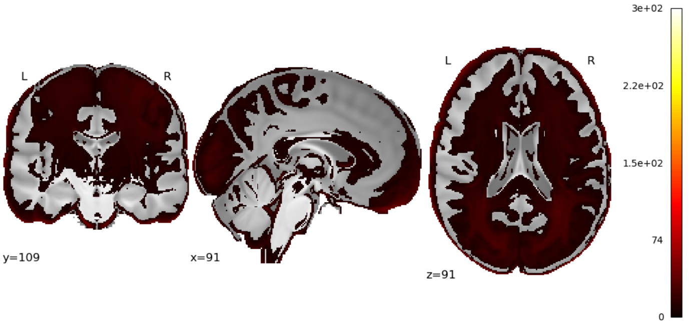

(i) PC 8

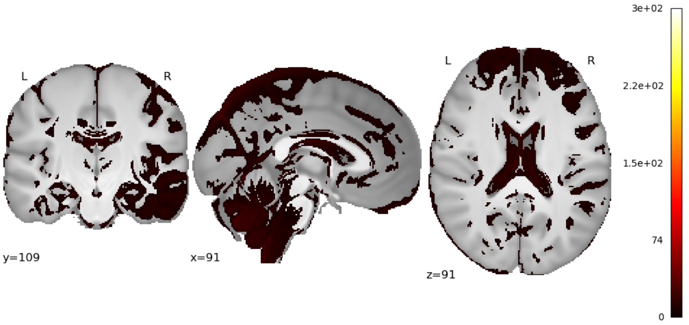

(j) PC 9

Fig G: **Statistical parametric mappings (SPMs) for the first 10 principal components of the model trained on the ADNI dataset.** Plotted are values of the t-statistics of the statistically significant correlation coefficients between between each PC and each single voxel in the MRI scans. We plot values below the Bonferroni-corrected significance threshold accounting for the total number of voxels tested.

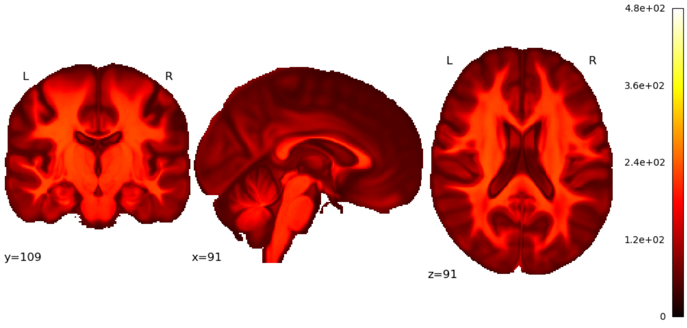

(a) PC 0

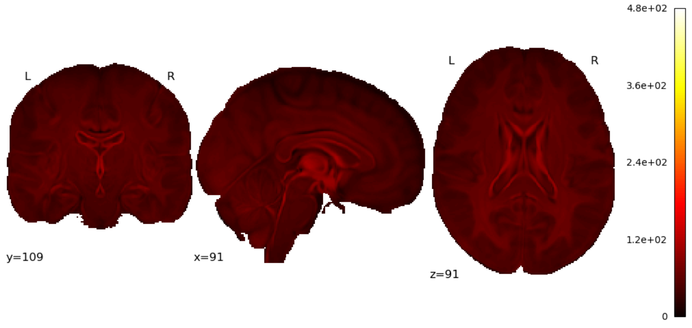

(b) PC 1

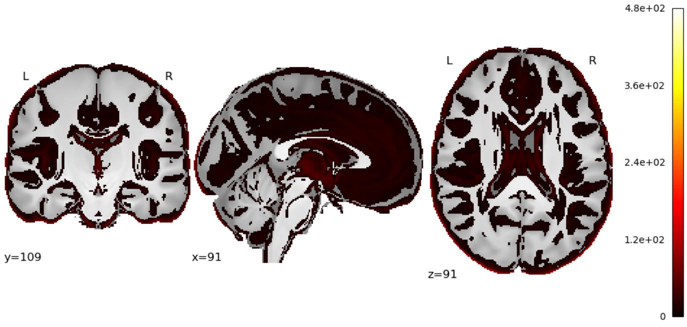

(c) PC 2

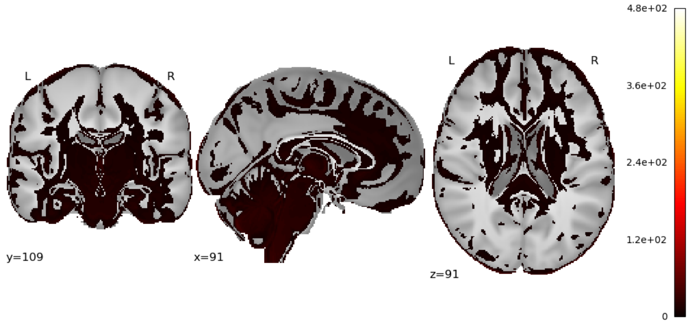

(d) PC 3

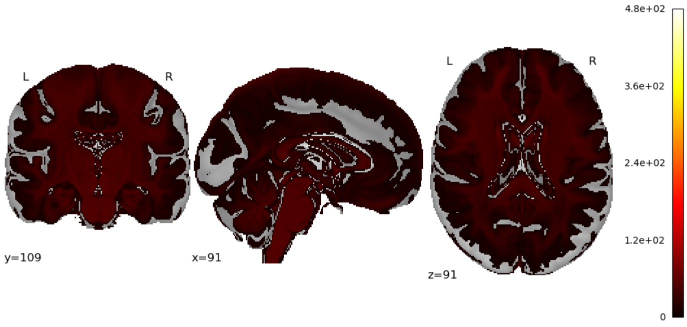

(e) PC 4

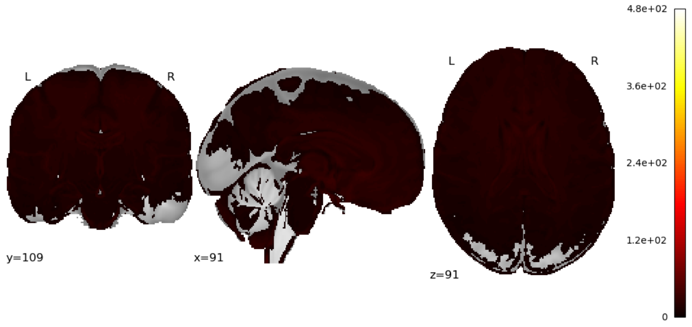

(f) PC 5

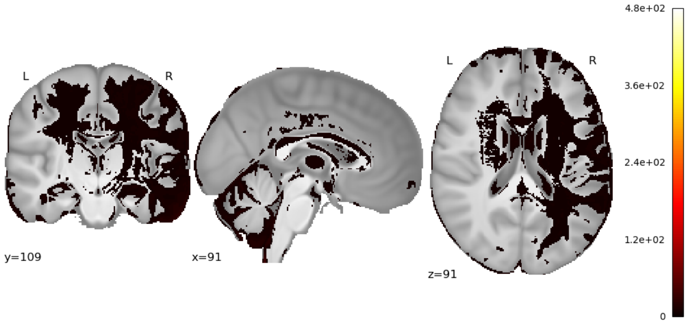

(g) PC 6

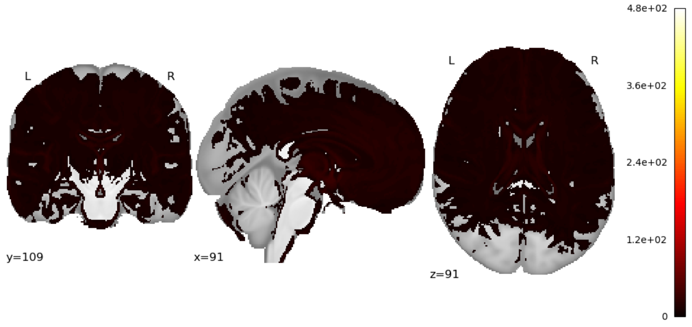

(h) PC 7

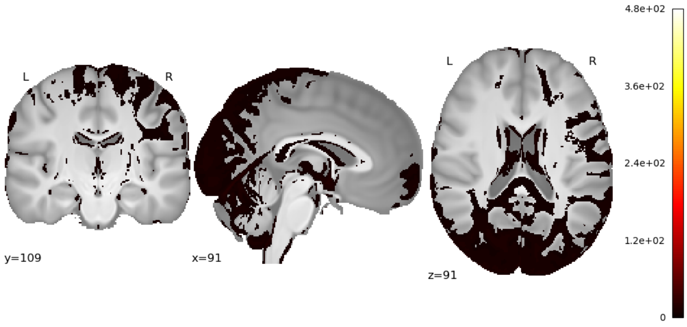

(i) PC 8

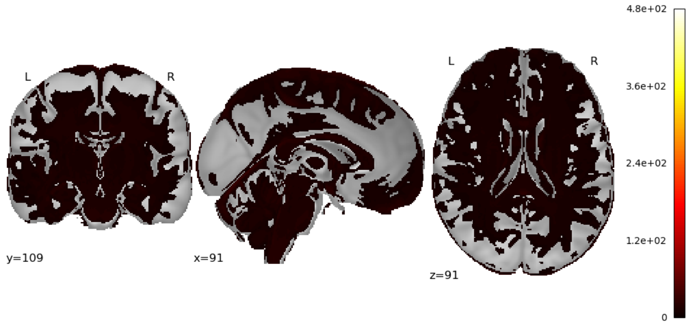

(j) PC 9

Fig H: **Statistical parametric mappings (SPMs) for the first 10 principal components of the model trained on the ImageNet dataset.** Plotted are values of the t-statistics of the statistically significant correlation coefficients between between each PC and each single voxel in the MRI scans. We plot values below the Bonferroni-corrected significance threshold accounting for the total number of voxels tested.

3.3 Correlations with Brain Regions

We computed segmentation masks for each brain MRI scan using the Synthseg software [1]. We grouped voxels into 19 ROIs, combining together regions corresponding to the left and right hemisphere in the appropriate cases. For each of the 20 PCs of the trained DNN models, we estimated the ratio of explained variance per voxel, and averaged the values over all voxels belonging to a specific brain region (Figure I). The ImageNet model explains a larger amount of variance than the ADNI model, in particular its first 2 PCs, which are significantly correlated with almost all voxels, indicating that the ADNI model is less focused on overall structural variation, and more on fine-scale changes, possibly due to the additional dementia classification objective it was trained with.

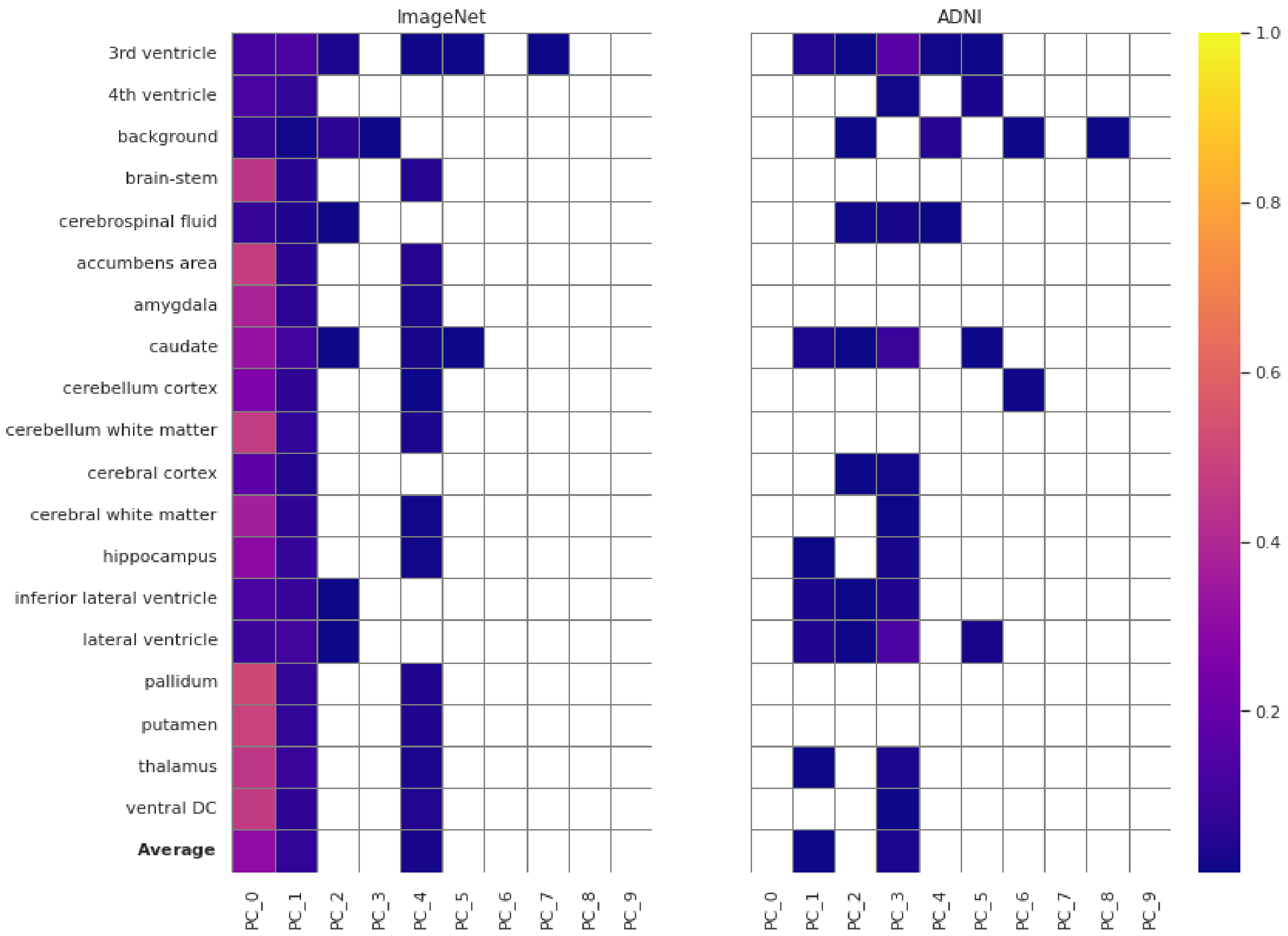

Fig I: **Ratio of variance explained in each brain region of interest (ROI) by each principal component of the neural network models features**. Values are computed as an average ratio over all voxels belonging to a ROI. Shown are only the pairs of PC – ROI with a ratio of explained variance higher than 0.01.

4 Additional Analyses of the Trained DNN Models

4.1 Correlations of ADNI Model PCs with Age and Sex

	PC_0	PC_1	PC_2	PC_3	PC_4	PC_5	PC_6	PC_7	PC_8	PC_9
Age	-0.03	0.28	0.01	-0.38	0.23	0.11	0.13	-0.07	-0.20	-0.10
Sex	-0.05	0.05	0.17	-0.40	-0.06	-0.27	0.17	-0.02	-0.12	0.08

Table B: **Pearson correlation coefficients of each of the 10 PCs of the ADNI-trained VAE model and age and sex covariates on the UKB data.**

4.2 Correlations Between PCs of the Two DNN models

In order to evaluate whether the two DNN models learned orthogonal information, we employed canonical correlation analysis (CCA) on the two sets of 10 PCs, with a maximum number of canonical pairs (10). The sum of squared correlation coefficients between each pair of canonical pairs was equal to 1.59 (i.e., 16% of total variance) indicating that the two models learned largely distinct features.

4.3 The Effect of Multi-task Training of the ADNI model on GWAS Results

In order to assess the gains of training the VAE model in a multi-task manner (reconstruction loss and dementia prediction), as opposed to the standard reconstruction objective, we trained a separate VAE on ADNI using only the reconstruction loss, and performed genome-wide association study (GWAS) using its PCs on the UKB sample. Compared to the $4,360$ distinct variants and 165 loci of the multi-task model, the "standard" model identified $1,997$ variants and 97 loci (out of which 87 were shared with the multi-task model), supporting the choice of the multi-task learning strategy.

References

[1] Benjamin Billot, Douglas N. Greve, Oula Puonti, Axel Thielscher, Koen Van Leemput, Bruce Fischl, Adrian V. Dalca, and Juan Eugenio Iglesias. Synthseg: Segmentation of brain MRI scans of any contrast and resolution without retraining. *Medical Image Analysis*, 86:102789, 2023. ISSN 1361-8415. doi: 10.1016/j.media.2023.102789.
