# Supplementary material for: TransferGWAS of T1-weighted brain MRI data from UK Biobank
Source: PLoS Genet. 2024 Dec 13;20(12):e1011332. doi: 10.1371/journal.pgen.1011332 (PMC11687923; doi:10.1371/journal.pgen.1011332)
Supplement: Attachment — Submitted filename: Brain_MRI_Transfer_GWAS_Response_to_Reviewers.pdf [file pgen.1011332.s006.pdf]

Response to Reviewers

We appreciate the questions raised by the reviewers. We have updated the manuscript accordingly, added a supplementary document with additional figures regarding several items mentioned, and address each individual point below:

Reviewer 1

R1 - 1: *"1. This study lacks too many details to clarify how the authors investigate the genetics of neuroimaging phenotypes, even though Figure 1 shows the workflow of this study. For example, in the Introduction, the authors mentioned: "We encoded the brain scans using 63 models pre-trained on the ImageNet [48] and Alzheimer's Disease Neuroimaging Initiative (ADNI) 64 datasets". However, it's unclear why they selected two datasets to train the data. Another example is that "As opposed to the ENDO approach.....". It's unclear how this ENDO approach works. What's the difference between ENDO and TransferGWAS. Hopefully, the authors could rewrite the Introduction and introduce more background for this study."*

We have rewritten the introduction in order clarify these, and other details. We hope the current version of the introduction can guide the reader better.

R1 - 2: *"In the Introduction, the authors mentioned that "Instead of using manually defined traits, a recent line of work employed deep learning (DL) to derive imaging features using pre-trained deep neural network (DNN)". Are there any references? What does "a recent line of work" refer to?"*

We have rewritten the introduction section - the works we are referring to in that sentence are now cited in the same sentence.

R1 - 3: *"In Section 3.1, the authors mentioned that "In order to interpret the signal carried by the DNN features, we extracted the first 10 principal components (PCs) of both DNN models". It's very confusing. What types of phenotypes did the authors use to extract the top 10 components? When did they extract the top 10, instead of the top 20 components? Since the authors put more Method details in the next section, they should introduce more background about how to confirm the top 10 components."*

We first extracted features from the 36,000 UKB brain MRI scans using the pretrained neural networks, yielding 2 times 36,000 deep features. We then performed PCA on these 2 sets of features, in order to reduce their dimensionality.

We agree that in the initial outline of the manuscript, referring to the principal components in Section 3.1 without earlier introduction could be confusing to the reader. We thus added an additional subsection (Section 3.1 in the updated manuscript) to the Results section briefly describing these steps.

R1 - 4: *"In Section 3.2, the authors tested the heritability of each PC and performed GWAS across all components, which is good. Based on Figure 4, I guess some of the PCs might not have significant heritability. So, it does not make sense to scan genetic variants associated with a trait that is not heritable."*

The heritability estimates for all the PCs were statistically significant, with the highest p value $< 10^{-8}$ (we have updated the manuscript to contain this information). We assessed the significance as per Finucane, H. K., Bulik-Sullivan, B., Gusev, A., Trynka, G., Reshef, Y., Loh, P.-R., ... & Price, A. L. (2015). *Partitioning heritability by functional annotation using genome-wide association summary statistics*. Nature Genetics, 47(11), 1228-1235.

R1: 5: *"I guess the authors performed GWAS for each PC. However, Figure 3 only showed two Manhattan plots for traits derived from ImageNet pretraining and ADNI pretraining. Where are the rest 18 plots? Could the authors explain it?"*

Figure 3 shows the Manhattan plots aggregated over all 10 PCs per model, taking the mininum p-value per SNP. We described that in the Figure's caption in the initial manuscript, but have updated the current version to make it clearer.

R1 - 6: *"In Section 3.2.3, the authors performed another GWAS using discovery and replication cohorts (23,604 and 12,709 samples). Where do these cohorts come from? Did the authors divide the overall sample into two parts? The authors are missing many details to clarify the results. Hopefully, the authors could add more details to the Results, which could help the readers better understand this study."*

These cohorts come from the same UKB sample on which we performed our main (full sample) GWAS. We updated the manuscript to include that information.

R1 - 7: *“In Section 3.3, the authors calculate the PGS for all remaining N = 451,450 participants not included in the GWAS sample. Because there are not many details in this section, I am not sure whether this cohort (N=451,450) is a European-ancestry sample. Since GWAS is generated from individuals with European ancestry, the PRS should also be generated in European ancestry samples.”*

We agree that it might be sensible to limit the evaluation of the PGS to the white-British sample, as per our GWAS. We repeated both the PheWAS and PGS predictive evaluation limited to the white British samples, and replaced the results in the main manuscript. We note that the PheWAS results yielded a visibly smaller number of significant hits, while the PGS results were almost identical (and even yielded more significant results). The previous results (computed on the whole UKB sample) were moved to the supplementary Section 2.

R1 - 8: *“Figure 6, I think it would be better if the authors showed these gene-brain associations in a 3D manner. The current 2D visualization could not clearly show these interesting findings, at least I could not see any significant brain regions associated with genetic variants. “*

We agree the initial plots were not easily readable. We have changed the way we plot these correlations (plotting the t-statistics for middle slices of each axis instead of automatically choosing the slice with the SPM plotting function) which should be easier to interpret now.

R1 - 9: *“ In the Section 5.2, SNPs were filtered with MAF≥ 0.1%. Since the study aims to investigate common genetic variants associated with brain phenotypes, common genetic variants are typically defined as MAF>1%. I would suggest the authors revise this MAF threshold to avoid false positive signals.”*

The applied filter of 0.1% MAF was not meant to limit us to only the common variants, but rather to filter out rare variants. While in standard GWA studies this might pose a risk of false postives, we note that we used BOLT-LMM, which should be more robust to false positives

The lack of inflation of false positives also seems to be confirmed by the QQ-plots (see Section 1 of the Supplementary material)

We note that the results were not strongly influenced by the variants with MAF < 1%: these were a total of 20 significant SNPs, out of which all but one were in loci with significantly associated SNPs with a higher MAF. The rs546521618 (6:156060806) variant with $p < 8 * 10^{-9}$ had no other variants in LD with it, and could be a potential false negative. We noted that in the revised version of the manuscript (Section 3.2.2). On the other hand, as can be seen in Figures 6 and 7 this variant (and others) were significantly correlated with a number (albeit small) of voxels in the brain scans.

The variants 7:120833182A_G and 12:49375064C_G are both in loci with other variants with MAF < 1% which are significant under the Bonferroni threshold, and with several common variants with p under 0.0001.

The other significant variants with MAF < 1% are in loci with at least one common variant below the Bonferroni-corrected threshold.

R1 - 10: *“In Section 3.3.1, the author performed PheWAS to screen for phenotypes associated with PRS. How did the authors perform phenotypic associations? What type of models did the authors use for testing associations? Given that UKB includes different types of variables (e.g., binary and continuous), do they use a single model for all phenotypes? The authors should add more details to clarify that.”*

As described in Section 5.3, we used the PHESANT tool [1] (https://github.com/MRCIEU/PHESANT), which is specifically suited for phenome scans in UK Biobank, and selects the appropriate model (linear, logistic, multinomial, or ordered logistic regression) based on the metadata of a particular phenotype field. We have updated the manuscript to include that information.

[1] Millard LAC, et al. Software Application Profile: PHESANT: a tool for performing automated phenome scans in UK Biobank. *International Journal of Epidemiology* (2017).

Reviewer 2

Major comments:

R2 - 1: “*The abstract and introduction of this work however do seem to state quite strongly the extent to which deep neural networks, and transfer learning are novel, and the extent to which voxelwise gwas is not done. The authors cite the ENDO approach of Patel et al. 2022. a) Aside from doing pretraining on a different dataset than the study dataset, in what was is TransferGWAS different from Patel et al. 2022? c) Methodologically, is the application of TransferGWAS in this paper the*

same as that in Kirchler et al. 2022? c) With regards to voxelwise, there are some pieces from early/mid 2010s such as from Thomas Nichols' group: "Fast and powerful genome wide association of dense genetic data with high dimensional imaging phenotypes" and Stein et al. "Voxelwise genome-wide association study". Are there really no updates to voxelwise GWAS since these works? And how does TransferGWAS compare to voxelwise?"

We have rewritten the introduction to address these points. In short:

a. transferGWAS differs from the approach of Patel et al. 2022 in that the training of the neural networks is performed on a separate dataset (or even modality), which allows to use the full size of the target dataset for GWAS

b. Yes, methodologically it is the same as in the original transferGWAS paper.

c. We are aware of updated to voxel-wise GWAS such as the MOSTest (Van der Meer et al. 2020, Shadrin et al. 2021), which additionally accounts for polygenicity. The main difference between GWAS on features extracted with neural networks and voxel-wise GWAS, is that the former can potentially capture complex non-linear dependencies in the data, whereas the voxel-wise approach is usually focusing on more linear changes wrt. each voxel

R2 - 2: *"The authors mention that they train the model for 500 epochs with the Adam optimizer. I understand that this was after pretraining on either ImageNet or ADNI (i.e., these are 500 epochs on UK Biobank. Pretraining on ImageNet has been found to improve accuracy of a resulting classifier, however that improvement can be lost if the length of training on the dataset-specific (target-dataset, in this case UK Biobank) is increased in terms of epochs. The question is this: Is the pre-training improving the nature of the local maximum that the backpropagation on the DNN eventually settles on? Or is it just getting us to the same local maximum (or an equivalent local maximum) faster? Can the authors please investigate this by increasing the number of epochs and plotting a measure of accuracy (or number of positives/amount of overlap of hits/or another unbiased measure of performance) per additional 100 epochs, for a few thousand more epochs? This would be greatly helpful to the community to show if TransferGWAS (or in general, any pretraining method) is truly bringing something that cannot be found when we do not pretrain, but train for longer. What are the compute costs? If 500 epochs takes a day, and if with two weeks of compute we can perform better than pre-training, then there is not much argument for pre-training (two weeks is not a long time)."*

We apologize for the confusion. We do not train (nor finetune) either of the models on UK Biobank, they are trained only on the auxiliary datasets. The reasoning behind is twofold:

- by only using a separate dataset for training, we can utilize the full sample size of the target dataset for GWAS
- using a different cohort allows to tailor the DNN features to a specific task, e.g., disease prediction (dementia status classification on the ADNI dataset), or extracting nuanced visual features (ImageNet training)

We have updated the manuscript to emphasize this distinction, and replaced "pretraining" with "training" in sentences where confusion might arise.

R2 - 3: *"It appears that the downsampling performed by TransferGWAS is quite extensive, downsampling to a side length of 96. Do the authors then introduce a brain mask within the 96×96×96 volume, to select only voxels that intersect the brain? This may considerably reduce the number of voxels that are then fed into the neural net, which could allow less extensive downsampling. I cannot find a mention of this in the paper, regrets if I missed it."*

As mentioned in Section 5.1, we performed brain extraction using the HD-BET tool (Isensee et al. 2019), followed by an additional non-linear registration to the MNI152 template.

Isensee F, Schell M, Tursunova I, Brugnara G, Bonekamp D, Neuberger U, Wick A, Schlemmer HP, Heiland S, Wick W, Bendszus M, Maier-Hein KH, Kickingereder P. Automated brain extraction of multi-sequence MRI using artificial neural networks. Hum Brain Mapp. 2019; 1–13. https://doi.org/10.1002/hbm.24750

R2 - 4: "*Can the authors comment on the clinical or scientific interpretation of the 14 claimed novel variants, and the type 2 diabetes link? I understand that this is not so much a methods paper, as TransferGWAS was already described in Kirchler et al. 2022. What scientific hypotheses regarding the mapping between genotypes, brain phenotypes and disease are supported or refuted by this work?"*

We describe the genetic correlations with type 2 diabetes in Section 4.3.1, and discuss the potential interpretations wrt. different dMRI traits, such as the non-trivial relation between fractional anisotropy of the left internal capsule and T2D.

For the potential novel loci, we investigated the correlations with brain regions for the lead variants of each loci (Figures 7 and 8), for the intron variants we described the brain (or bone density) associations of their corresponding genes, and

analyzed tissues for which the remaining loci were marked as enhancers (Section 3.3.2).

R2 - 5: *"While early work on brain imaging in UK Biobank often focused on the white British subset, the sample size is now large enough that meta-analysis or replication analysis with ancestries other than white British can now be done. How generalizable to ancestries other than white British are the results? An answer to this question is essential, in order to prevent bias from sample inclusion criteria."*

While the sample size of the **whole** UK Biobank might allow for replication analyses on non-white British population, we note that the sample size of participants with a brain MRI scan is over an order of magnitude smaller (around 40,000). We excluded 6,484 participants with the UKB field 22006 set to false (not self-identifying as white-British or not genetically similar). However, the majority of this remaining sample is still of European descent, both from self-identified ethnicity and from genetic ancestry estimates based on 1000 genomes (see the two tables below). The largest non-european ancestry, CSA is below 500 samples, what, according to our judgment, is not large enough for conducting another GWAS. We also note, that the similar GWA studies on brain MRIs from UKB that we compare against (Smith et al. 2021, Patel et al. 2022) were also done on the white-British subset.

Self-identified ethnicity:

Ethnicity	Count
British	2652
Any other white background	1318
Irish	1097
Indian	301
Other ethnic group	231
Caribbean	162
Chinese	124
African	119
Prefer not to answer	94
Any other Asian background	71
Pakistani	69
White and Asian	62
Any other mixed background	60
White and Black Caribbean	44
White and Black African	26
White	21
Bangladeshi	7
Do not know	5
Any other Black background	3
Mixed	2
Asian or Asian British	1

1000 Genomes estimates:

Ancestry	Count
EUR	4024
CSA	491
AFR	254
EAS	185
AMR	118
MID	51

R2: 6. *"Please refer to inclusion criteria based on ancestry in a consistent way ("white British")"*

We have updated the manuscript accordingly

Minor comments:

R2 - 1:. *"Please increase the font size of Figure 5, possibly after rotating it 90 degrees clockwise. Increase font size of Figure 6, after tiling in two columns. Greatly increase font size of Figure 3, possibly tile in one column."*

We have updated the figures accordingly. We did not rotate Figure 5 however, as this would require displaying the trait names vertically, which we find less readable.

R2 - 2: "*Please refer to UK Biobank as "from UK Biobank" rather than "from the UK Biobank". "*

We have updated the manuscript accordingly.

R2 - 4: *"In the fourth sentence, the em dash should be longer"*

We updated the manuscript accordingly

R2 - 5: "*The references need formatting attention. For example, "early alzheimer's disease" → "early Alzheimer's disease" and "Nature genetics" → "Nature Genetics" and "The relationship between bmi and onset ..." → "The relationship between BMI and onset ..." and "PLoS genetics" → "PLOS Genetics""*

We have updated the manuscript accordingly

R2 - 6: *"The tables in the supplementary material could do with captions indicating units, and describing in more detail what the columns mean. I struggled to read the supplementary tables."*

We have updated the tables with appropriate column descriptions.

Reviewer 3

R3 - 1: *"I believe the "Interpretation of DNN Features" Section should be improved and enlarged. While the presence of many significant correlations with different traits is shown in an aggregated way in Fig. 2 and the individual correlations are included as Supplementary Material, the question of what's the impact of the different embeddings in brain morphology is central and should be addressed. I would suggest distilling the conclusions from the correlation study into a couple of additional paragraphs, so that the reader can better understand what each of the 20 PCs is modelling. An additional figure (e.g. another panel in Fig. 2) showing the correlation of some brain structural traits with the PCs could perhaps help to this end."*

We have added a figure summarizing correlations with brain regions (Figure 3), along with a range of figures in the Supplementary Section 3 (please also see answer to question 2 below), and added a paragraph summarizing these findings in the main text (Section 3.2).

R3 - 1 [2]: "*While some correlations seem to be described in Section 3.4 "Genetic correlations", it's not clear if these are genetic correlations (which the section title suggests) or "direct" correlations (as the text suggests). In any case, I believe Section 3.1 would be a better place to include this information."*

We have corrected the text to make it clearer when we are referring to genetic correlations.

R3 - 2: "*It would also be reassuring to determine whether these insights are confirmed by directly examining the impact of the DNN-derived PC embeddings in the image space. For example, in the case of the VAE network, traversals can be performed on the PCs, e.g. from (mean - 3 sd) to (mean + 3 sd), and examine changes in the output of the decoder. This could help localize the changes to some specific region of the brain. The PCA inverse transformation could be pre-appended to the decoder network as a fully connected layer, so that your DL code can be readily used to create these figures. In the case of the ImageNet, since you have no decoder you could try to build attribution maps by employing explainable AI techniques. For example, if using Pytorch the library Captum might be handy to this end. The average operation can be added as an additional Pytorch module, and Captum could be used on a 3D model built in this way."*

We added an extensive section in the supplementary material (Section 3) investigating each of the 20 DNN PCs. We decided not to use off-the-shelf interpretability methods, which in our opinion are not straightforward to use, as they involve choosing a range of hyperparameters, which are not trivial to tune (e.g., the variance of noise in GradCAM methods), and are usually developed with 2D data in mind.

Instead, we leveraged the fact that the scans are aligned to the MNI template, which allows us (to some extent) to analyze them on a per-voxel level. While this approach might miss certain non-linear relations, we found the generated explanations

easier to interpret. On experimented with using gradient-based saliency methods, but found them to often yield noisy heatmaps, even with extensive hyperparameter tuning.

R3 - 3: "*In Fig. 2, please clarify if you are counting a single association whenever you have more than one significant PC-trait association with a given trait. I understand this is the case since you refer to these values as fractions, however the caption seems to suggest otherwise (i.e. "we plot the number of significant associations per model" this reads as though there could be double-counting). Also, could more fine-grained results be presented, showing whether"*

Yes, we are only counting a single association in that case, we have updated the caption to avoid future confusions.

We note that in the review we received this comment seems to be cut:

"Also, could more fine-grained results be presented, showing whether [END]"

R3 - 4[1]: "I*s there an expected benefit in training the VAE in a separate dataset instead of directly on the UK Biobank population? Is this done because the ADNI dataset contains a greater number individuals with brain pathologies which can be used to guide the training?"*

The main reason behind training on a separate dataset is being able to use the full sample size of the target (UKB) dataset for GWAS. The additional potential benefit, as mentioned by the Reviewer, is being able to specialize the DNN-learned features for a particular task, e.g., pathology detection, which was precisely our case - ADNI is well suited for training dementia prediction models, as it contains demographics-matched cases and controls, whereas UKB contains a relatively low number of (detected) dementia cases (and in general represents a healthier sub-population than the general one [1]).

Anna Fry, Thomas J Littlejohns, Cathie Sudlow, Nicola Doherty, Ligia Adamska, Tim Sprosen, Rory Collins, Naomi E Allen, Comparison of Sociodemographic and Health-Related Characteristics of UK Biobank Participants With Those of the General Population, *American Journal of Epidemiology*, Volume 186, Issue 9, 1 November 2017, Pages 1026–1034, https://doi.org/10.1093/aje/kwx246

R3 - 4[2]: "*Also related to this, I am curious as to whether the authors have assessed the heritability of features that come from a VAE trained simply with the reconstruction task. The conclusion that a benefit is found in using multi-task learning with a prediction head would be really interesting to report.***"**

This is indeed an interesting analysis to include. We have now trained another VAE with just the reconstruction task, and performed the same GWAS on its PCs, which yielded 97 independent loci (87 of which were shared with the multi-task model), compared to the 165 loci of the original, multi-task model reported in the manuscript. We have added these results to Section 4.3 of the Supplementary.

R3 - 5: *"I like the idea of inputting demographic data to the decoder to "explain them away" from the embeddings. The correlation of the embeddings (or the PCs) with age and sex can be reported to ensure that this procedure is having the expected effect."*

We report the correlations in Section 4.1 of the Supplementary Material. We note that several PCs are moderately correlated with either age or sex (e.g., PC_3 with a correlation coefficient of ~0.4 for both sex and age).

	PC_0	PC_1	PC_2	PC_3	PC_4	PC_5	PC_6
**age**	-0.03	0.28	0.01	-0.38	0.23	0.11	0.13
**sex**	-0.05	0.05	0.17	-0.40	-0.06	-0.27	0.17

R3 - 6: *" I wonder if the data matrix for PCA in each of the two experiments, consists of hidden features that are properly scaled. In principle, the neural network training could converge to a set of hidden features that span different ranges. For instance, hidden feature h1 could span the range [-3, 3] and h2 could span [0.1, 0.2], where the amplitude of these ranges is not necessarily meaningful and which would affect the results of PCA. In particular, some hidden feature with a large range can be overshadowing another, more important one, but with a smaller range. It would be reassuring to know this is taken into account somehow, either by scaling the hidden features to a common range or by justifying why this is not actually desirable."*

We agree that the appropriate scaling should be taken into consideration. In the case of the VAE model, the imposed standard Gaussian prior is regularizing the spread of active latent space dimensions to prevent drastic outliers (please see the plot below for a histogram of empirical standard deviations of the latent space dimensions). As for the ImageNet trained ResNet model, we took outputs of a hidden layer which applies Batch Normalization (followed by ReLU), which should results in roughly similar feature scales.

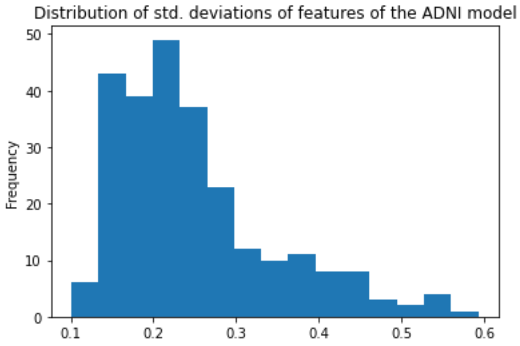

R3 - 7: *" The choice of 10 as the number of PCs to be considered should also be justified, e.g. in terms of the proportion of variance explained or the absence of GWAS hits."*

We decided for 10 PCs similar to Kirchler et al. 2022, who found that a larger number led to a minor improvement in discovered associations, and due to the fact that the 10 PCs accounted for 92% and 81% of variance in the ImageNet and ADNI trained models respectively. We updated the manuscript accordingly (Section 3.1).

R3 - 8: *"It would be interesting to examine how PCs from the two different models relate to each other. In particular, are they linearly identifiable, i.e. can a linear transformation be applied to one set of PCs to obtain the other? Canonical correlation analysis could be helpful to this end. If the answer is no, i.e. if some PCs can't be accurately reconstructed as linear combinations of the other set of PCs, this would provide evidence that the two networks actually extract orthogonal information, further justifying the use of both."*

This is indeed an interesting analysis, and we included it in the Supplementary Section 4.2. We computed CCA between both sets of PCs, and found that they are largely distinct, as the norm of the cross-covariance matrix of the canonical pairs was equal to 1.59 (where a value of 10 would indicate that both sets of features encode identical information).

R3 - 9: *" In the abstract, I suggest adding a closing statement regarding the possible implications of the findings."*

We have updated the manuscript accordingly

R3 - 10: *" For Table 1: is a statistical test being used to declare the improvement significant? If so, what's the test? If not, I would discourage the use of the word "significant" since it normally implies a statistical test being employed, at least in my experience.*"

As described in Section 5.4, we employed pairwise permutation tests with 1,000 permutations to compute p-values for differences in performances of each PGS. To avoid future confusion, we have added this information to the table caption as well.

R3 - 11: "*Line 337: Usually, a threshold in INFO imputation score is applied (typically 0.3). Is this filtering step being performed here? It looks to be the case based on the number of SNPs, but it would be good to explicitly mention it.*"

We did not explicitly exclude variants based on the INFO score when performing GWAS, however none of the significantly associated variants had an INFO score below 0.5.

R3 - 12: *"For Fig. 3, I suggest adding gene names. Given the large number of loci and the central role of these figures, also consider using landscape orientation and splitting the Manhattan plots into two different figures. It would also be informative to add some indication for the GWAS hits of whether the loci are shared between the two models, or whether effect sizes are significantly higher in one case or the other."*

We have split the Manhattan plot in two rows for better readability and added gene names. As per the suggestion, we updated the manuscript specifying the number of loci shared between the two models (Section 3.2), and included a per-chromosome overview in the form of Venn diagrams in the Supplementary Section 1.2.
